# Reflections: NIAAA’s Directors Look Back on 25 Years

**Published:** 1995

**Authors:** Susanne Hiller-Sturmhöfel

**Affiliations:** Susanne Hiller-Sturmhöfel, Ph.D., is a science editor of Alcohol Health & Research World

**Keywords:** government agency, interview, public policy on AOD, AOD dependency, AOD abuse, research

## Abstract

During its 25 years of existence, the National Institute on Alcohol Abuse and Alcoholism (NIAAA) has been influenced by many individuals and organizations. Among those shaping NIAAA’s missions and their implementations, the Institute’s directors played and continue to play a pivotal role. In return, the position as director of NIAAA had an impact on these men and their careers. *Alcohol Health & Research World* contacted the former and current directors to record their personal recollections and impressions of the alcohol field and NIAAA’s role in it.

## Morris E. Chafetz, M.D

### Director, 1971–1975

***AH&RW:*** Dr. Chafetz, how did you get involved in the alcohol field? It was not really a popular field in the 1960’s and 1970’s.

***Chafetz:*** No, it certainly was not. What got me into the field on July 1, 1954, was pure opportunism. I had finished my training at Harvard Medical School and Massachusetts General Hospital and there was no job available except one. The State had given Massachussetts General Hospital money to start an alcoholism clinic, and no other psychiatrist would take the job. I did not think much of alcoholic people. I did not like them; I just was not the least bit interested in them. But I loved the hospital and I wanted to stay there. And it only took me a few months of listening to these patients to recognize my prejudices and the prejudices of others. I realized that this issue reflected every social health policy problem being faced by the country. This realization is why I have enjoyed working in the field for 41 years and still enjoy it.

***AH&RW:*** Before you became the first director of the National Institute on Alcohol Abuse and Alcoholism (NIAAA), were you also involved in the lobbying process for the legislation that established the new Institute?

***Chafetz:*** Well, I testified before Senator Harold Hughes’ committee when it was preparing the legislation in 1970.^1^ I represented the American Psychiatric Association. As you can imagine, there were very few people who were interested in the legislation. The only people who were really interested in the issue were people who had had alcohol problems themselves or in their family. I didn’t qualify for that.

**Figure:**
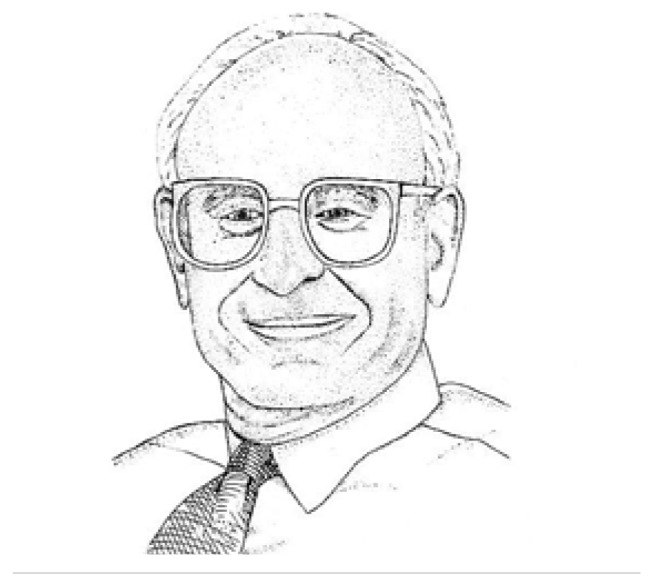
Morris E. Chafetz, M.D., was NIAAA’s first director. After leaving the Institute, Dr. Chafetz founded the Health Education Foundation, an organization that focuses on the relationships between lifestyles and health. He still serves as the president of the foundation. In addition, Dr. Chafetz served on President Reagan’s Presidential Commission on Drunk Driving.

But people who have suffered an illness tend to be subjective and judge the problem only from their experiences. Because I was not personally affected by alcoholism, I could take what I felt was a more objective perspective. For example, I remember that when Marty Mann^2^ first heard me speak in 1959, she said, “I disagree with everything that you’ve just said, but for the sake of the field, please keep saying it.” Also, when I testified, Senator Hughes—who himself had recovered from alcoholism—asked me some critical questions and he was startled by my candor in responding to them. We became fast friends after the hearings.

In September 1970 I took a position in what was then the Division on Alcoholism at the National Institute of Mental Health (NIMH), so officially I could not lobby for the alcohol legislation. But that didn’t mean I did not quietly work very hard to have it passed. On December 18, 1970, I was in the gallery with my wife when the bill was passed in the House of Representatives, and I went down and hugged Congressman Paul Rogers who had steered it through. I later learned that I should not have been present when my bill was being considered. But I was just too ignorant of all the rules and regulations to have known that.

Everybody then said that President Richard Nixon would never sign the bill because he was very much opposed to it. I was going out of the country but I reassured all my friends that “Chafetz luck” would operate, and President Nixon finally did sign the bill on December 31. I later was told that he signed it without a word.

***AH&RW:*** What was it like to be in charge of establishing the new Institute?

***Chafetz:*** Well, as Harold Hughes said to me, I had a one-in-a-million opportunity. I had spent 16 years studying alcoholism in an academic setting; I had been all over the world studying the problem. I really was quite immersed in it. When I was invited to become the director, I couldn’t pass it up, it was just an unbelievable opportunity. Who gets the chance to start a Federal agency in their area of expertise, the field they have studied for years? It really was the greatest 5 years of my life.

***AH&RW:*** What was NIAAA’s most important mission in that initial period?

***Chafetz:*** At that time, it was to get rid of the stigma that was associated with alcoholism. I remember that Harold Hughes could not recruit a public figure to testify about his or her alcohol situation. Finally, we convinced [actress] Mercedes McCambridge to speak. She put her career in jeopardy by testifying. Now it has become almost the other extreme. It is almost fashionable to have an alcohol problem and to say you are recovering from it. But at that time, alcoholism was so stigmatized, was so looked down on.

Having experienced the extent of my own prejudices and my own ignorance of the issue, I was bound and determined to turn the country around and to treat alcoholics as ill human beings who needed treatment, not as bad people who should be ignored and neglected. I remember saying in one of my first speeches that alcoholism was America’s most treatable, untreated illness, and I still feel that way. But the Institute also became involved in a lot of other areas. We got into prevention, we got into international programs.

When I started at NIAAA, the budget was $6.5 million. By fiscal year 1974, my last year at the Institute, we had a staff of only 90 employees, including secretaries, but a budget of $214 million. At that time, we funded everything: grants for the State programs, education, prevention, treatment, international work—you name it, we did it.

***AH&RW:*** How do you think NIAAA’s mission has changed over the years?

***Chafetz:*** I have studied the history of alcoholism and alcohol research, and I have repeatedly seen a shift in focus away from the people and onto the drug alcohol. I think that this is an unfortunate situation. I consider alcoholism and alcohol problems a people issue, a societal issue.

This shift of focus from the disease alcoholism to the drug alcohol is what bothers me the most because alcohol itself really does not count: The issue is people. I am a people person in all my activities. That is why at NIAAA we started a prevention program that focused on responsible drinking. That is why the foundation I head now has developed a program with the acronym TIPS [Training for Intervention Procedures by Servers of Alcohol], which teaches people around the drinker to make sure that the drinker does not get into trouble. I believe in local education; I believe in responding to human beings. But I have a feeling that some of the missions of the Institute got distracted away from the people.

***AH&RW:*** Do you think that the individual alcoholic or health care professional benefits from all the scientific advances of alcohol research?

***Chafetz:*** My view of alcoholism is that it is a multidimensional illness that involves many facets—genetics, physiology, societal aspects, and so on. Although research has refined some of these specific aspects of alcoholism, I don’t believe that we have made the progress necessary to help us understand or successfully treat alcoholism. It is such a multidimensional problem. We still do not have clear definitions of alcoholism and alcohol problems. How can we measure the outcome of a genetic predisposition if we do not have a clear definition of the disease? Alcoholism is not the kind of condition that lends itself to easy conclusions.

***AH&RW:*** What do you think has been NIAAA’s most outstanding achievement over these 25 years?

***Chafetz:*** The most important achievement, in my opinion, is that NIAAA has made the country recognize that alcoholic people are not bad but are ill and in need of treatment. The social stigma has been removed and people now will seek help. There are still many things missing in the system of providing help, but it is nonetheless an enormous success. Imagine breaking down such a prejudice in only 25 years! That is a remarkable achievement.

***AH&RW:*** What has your tenure at NIAAA meant to you personally, and how has it affected your career?

***Chafetz:*** For one thing, it brought me to Washington, DC, and I have stayed ever since. And the time at NIAAA certainly was the greatest professional experience of my life. I could not have been luckier than to have had that experience of founding a Government agency.

The experience as Institute director also gave me a new perspective—that no matter how wonderful treatment was, it could only contain the flood of alcohol problems, not stop it. I realized that we had to move to a prevention modality. That realization motivated the prevention program for responsible drinking at the Institute, and it was the reason why I later developed the TIPS program. I think that to teach people in bars, in universities, in grocery stores, and in the workplace, to give them the tools to intervene and prevent alcohol problems, is the way to go. Prevention is the key and will always be the focus of my activities. And when I talk about prevention, I don’t mean lecturing or putting out slogans and signs. I am talking about people having an impact on other people. That is how true changes in behavior are made. My work in prevention gives me almost as much satisfaction as I experienced starting the Institute.

After I left NIAAA, I also was appointed by President Ronald Reagan as the chairman of the Education and Prevention Committee of the Presidential Commission on Drunk Driving. That was a great experience as well.

***AH&RW:*** What direction should the alcohol field take over the next 25 years in your opinion?

***Chafetz:*** I would hope that we would acquire a better understanding of how we can prevent alcohol problems. And rather than focus on the drug alcohol, I would like to see a focus on people. The research should be on people, not on a substance that has been around since the beginning of time.

## Ernest P. Noble, Ph.D., M.D

### Director, 1976–1978

***AH&RW:*** Dr. Noble, can you describe briefly how you got involved in the alcohol field?

***Noble:*** After receiving my Ph.D. in biochemistry, I went to medical school at Case Western Reserve University, where I had some contact with alcoholics as a medical student. However, I really began to appreciate the problem as a resident in medicine at Stanford University. There, for the first time, I saw the vast devastation that alcohol caused in the patients. At that time, the diagnosis rarely was alcoholism; doctors called it liver disease, they called it heart disease, or they called it psychosis. But I saw that the common denominator among the patients was alcoholism, that many of them were hospitalized for alcohol-induced problems.

After I finished my residency and became an assistant professor in psychiatry at Stanford, I felt that I needed more training in basic neurosciences. With a career development award from NIMH, I went to the laboratory of Dr. Julius Axelrod, the future Nobel laureate, who got me very excited about neurotransmitter systems. When I returned to Stanford, the problems I saw in the alcoholics and the possibility that alcohol might affect the brain through the neurotransmitter systems made me think that this would be an interesting area to research. Many of my colleagues at Stanford and elsewhere tried to discourage me, suggesting that I should pursue more exciting areas, such as schizophrenia or depression. However, I felt that I wanted to get into the alcohol field and began to study the genetics of alcoholism in animal models, using mice that either did or did not prefer alcohol.

A few years later, while I was taking a sabbatical in Strasburg, France, I received a call saying that Dr. Morris Chafetz, the first director of NIAAA, had resigned and would I be interested in the position. I thought, “Why should I? I’ve got a very good job, I earn a good salary, and I live in a beautiful area in California,” so I discouraged the first caller. But then I got a second and third call, which showed me that this was a serious offer. Finally my wife said, “Why don’t you put your hat into the ring and see what happens.” So I did. A few weeks later, I was called to Washington, DC, for an interview and selected to be the director. That is how I came to NIAAA.

***AH&RW:*** During your time at NIAAA, what did you consider the Institute’s most important program, most important mission?

***Noble:*** I thought two fundamental issues had to be addressed. The first was to reduce the devastation caused by alcoholism in our country. The second, which I had more personal experience with, was to create a substantial base of research and knowledge about alcohol’s and alcoholism’s effect on the body systems.

When I became NIAAA director, the per capita consumption of alcohol was higher than it had ever been in the history of our country, even higher than just before Prohibition. As a biochemist and biologist, I knew that alcohol is a cytotoxic and addictive drug, and I saw a strong connection between the amount Americans drank and the kinds of alcohol problems we were faced with. This was not just my personal point of view, nor was it limited to the United States. We did a thorough study of alcohol consumption around the world and everywhere found a very close relationship between the amount of alcohol consumed and the problems that alcohol engendered. Therefore, I felt that we had to develop measures to decrease alcohol consumption. This is, of course, also a political issue, because alcohol is a socially accepted beverage. It is a big industry, and consequently, just the concept of reducing consumption created conflicts.

**Figure:**
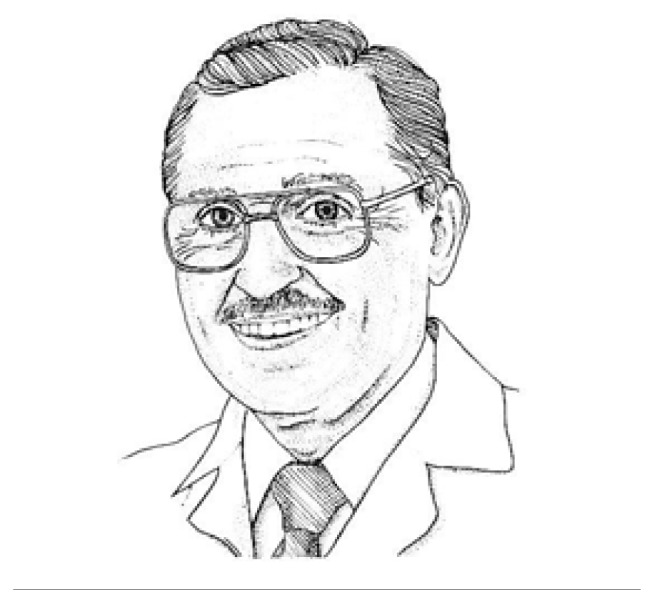
After serving as director of NIAAA, Ernest P. Noble, Ph.D., M.D., was appointed associate administrator of the Alcohol, Drug Abuse, and Mental Health Administration (ADAMHA; now called the Substance Abuse and Mental Health Services Administration). Since 1981 he has been Pike Professor of Alcohol Studies and director of the Alcohol Research Center at the University of California, Los Angeles (UCLA). He also has served as the director of the Alcohol and Drug Treatment Program at UCLA since 1983.

I felt that we should use the public health model as a means to mitigate the alcohol problems in this country. This model entailed that to diminish alcohol consumption, we had to consider three factors: the host (i.e., the drinker), the agent (i.e., alcohol), and the environment (i.e., social attitudes toward drinking).

Along that same line, in 1977 I first addressed fetal alcohol syndrome (FAS) in a message to the Nation. FAS captured the imagination of people—it was a problem we could prevent, and it demonstrated for the first time that the agent alcohol itself caused the damage. The efforts to prevent FAS have progressed rather rapidly since then. For example, research grants were awarded by NIAAA to study FAS, and warning labels were introduced, not only on [alcoholic beverage] bottles but also in bars in some States. Prevention of FAS was one project that took off right away and that has become one of NIAAA’s most important successes.

As for the second fundamental mission, enhancing the base of knowledge, we were able to double the research budget for the Institute. We also established 9 alcohol research centers throughout the country, which now have been expanded to 14 centers. Those programs have been very successful in increasing the knowledge about alcohol.

***AH&RW:*** Are there other areas in which you feel NIAAA has made important contributions in the past 25 years?

***Noble:*** I think the public health model was one of the most important ones, and there were successful spinoffs, for example, in terms of drinking and driving. Once the people’s consciousness was raised about the damage that alcohol can induce, grass roots movements began to sprout, such as Mothers Against Drunk Driving, which were seeded by NIAAA and which have become quite successful around the country. As a result, the numbers of alcohol-related highway deaths and injuries have decreased significantly.

Another success of NIAAA is the destigmatization of the alcohol problem. As I mentioned earlier, during my residency at Stanford, we did not make a diagnosis of alcoholism because of the shame that it would bring to the patient. Today the identification of alcoholics is much more successful than it was then. The stigma is decreasing, although it still exists; a small but significant segment of the population still considers alcoholics as weak-willed, morally depraved individuals. The destigmatization has been achieved through NIAAA’s efforts in alliance with other organizations, such as the National Council on Alcoholism (NCA). It is, I think, due in part to research findings that show a strong genetic component to alcoholism. If there is a genetic predisposition to alcoholism that people inherit, then it is not necessarily their fault if they develop the disease, and we should show them more compassion.

***AH&RW:*** Do you think the individual alcoholic or health care professional benefits from the scientific advances in the alcohol field?

***Noble****: *I am sorry to say that the actual application of research knowledge to treatment is still lagging behind. Besides raising the awareness of FAS and drinking and driving issues, we have not made much progress in affecting alcoholics and the care they receive. After talking to many health care professionals around the country, I have the impression that the research information that has been gathered has not been transferred successfully from the so-called bench to the bed. There really have not been any significant practical spinoffs for treatment. We still have few effective pharmacological agents, and alcoholics now are treated pretty much as they were 20 or 30 years ago. I think there has not been the kind of significant progress in the treatment of alcoholism as in areas such as heart disease during the past decade.

***AH&RW:*** What do you consider the most important alcohol-related questions for the next 25 years? Where is the greatest need for improvement?

***Noble:*** I think the greatest need for improvement is in really understanding what this arcane disorder called alcoholism is all about. We need to understand better the contribution of the environment to the disease; we need to understand better the contribution of genetic factors; and, perhaps most importantly, we need to understand better how these factors interact to cause alcoholism in some people or to protect other people from it. That is a fundamental question, and the only way to answer it is through research. People might not be pleased with all of the research results, but we need objective information instead of being moved by our own feelings about what the disorder alcoholism is.

***AH&RW:*** What was the most important impact that your tenure as NIAAA director had on you and your career?

***Noble:*** Being director of NIAAA was a very exciting and highly rewarding experience. Before I came to NIAAA, I was a scientist, so I mainly was involved in research and had limited experience in the treatment of alcoholics. As NIAAA director, I suddenly was thrust into a national, even international, limelight. I was expected to understand, or at least to look at, the whole issue of alcoholism from a panoramic standpoint. I was the number-one Federal representative on whose shoulders was the responsibility to do something about the problem. Therefore, I wanted to really understand the problem first hand. I traveled across the Nation; I went to virtually every State including Alaska; I went to the small villages; I went to the ghetto areas, the black areas, the Hispanic areas, the poor white areas; I went to women’s programs, children’s programs, and adult programs. I got a first-hand view of the devastation that alcohol causes and of what was being done to deal with it. This exposure to the whole range of problems that alcohol created in our Nation was very beneficial for me and expanded my own narrow point of view about alcoholism.

Another personally rewarding aspect was the interaction with Congress and the White House. I tried to use the knowledge that we had to educate those in higher positions about the problems that alcohol caused. As NIAAA director, I had to be a spokesperson; I felt that at least I had to put forth my ideas. Some people may not have liked what I was saying, but it had to be said so that we could educate people about the importance of alcohol programs, so that we would get more funding and more attention to this number-one drug problem in America.

## Loran Archer

### Acting Director, 1978–1979, 1981–1982, and 1986

***AH&RW:*** Mr. Archer, when did you start working at NIAAA and how did you become involved in the alcohol field?

***Archer:*** I came to NIAAA in the summer of 1977. But I initially became involved in the field in 1956, when I came out of graduate school, trained as a vocational rehabilitation counselor. My first assignment was to work as a liaison for vocational rehabilitation in a Veterans Administration hospital and a county tuberculosis (TB) sanitarium. In the TB clinic, I discovered quickly that many patients were alcoholics. I became very interested in that, because these people came from a wide variety of backgrounds and were not the “skid-row” alcoholics I typically had thought of.

Later I served in Fresno, CA, on the board of the local TB association. At that time, the TB association had taken the recently established NCA under its wing, assisting the council in getting started in California. That was when I first met [NCA founder] Marty Mann and became very interested in her projects.

Around the same time, the national interest in alcoholism was growing, and a joint liaison at the national level was established between vocational rehabilitation and the alcoholism programs. California also created such a position, and I became the liaison to the California Department of Public Health, which was responsible for the alcohol programs. There I was influenced by Dr. A.C. Hollister, an epidemiologist who headed California’s alcohol program. I held that position for a little over a year and helped develop a specialized vocational rehabilitation program for alcoholics. The responsibility for alcoholism treatment programs subsequently was transferred completely to the vocational rehabilitation department, and I became the director of California’s alcoholism prevention and treatment programs. That was probably one of the most exciting and educational times for me, because I had complete freedom to pursue some innovative ideas. For example, in an effort to reach employed clients, we established treatment programs that stayed open in the evenings.

In 1977 Dr. Ernest Noble, who had become director of NIAAA in 1976, invited me to come to the Institute to work as an executive assistant with him. When Dr. Noble left the Institute in 1978, I became the acting director. That is how I became involved with NIAAA, which is a somewhat strange way. But I think people came into the alcohol field from a wide variety of directions.

***AH&RW:*** That was the first of your three tenures as acting director?

***Archer:*** Yes, I was actually never director, always acting director. But I was very fortunate, because each time I received very strong support from the administrators of ADAMHA^3^ and thus had good authority. Also, I discovered that the acting director tended to stay at the Institute, whereas the directors left to take on other positions. And because I enjoyed being at NIAAA and wanted to continue working in the alcohol field, I was pleased to stay in my position as deputy director.

***AH&RW:*** After your lengthy experience at NIAAA, what do you think are the unique characteristics that have enabled the Institute to spearhead the advances in alcoholism research, treatment, prevention, and so on?

**Figure:**
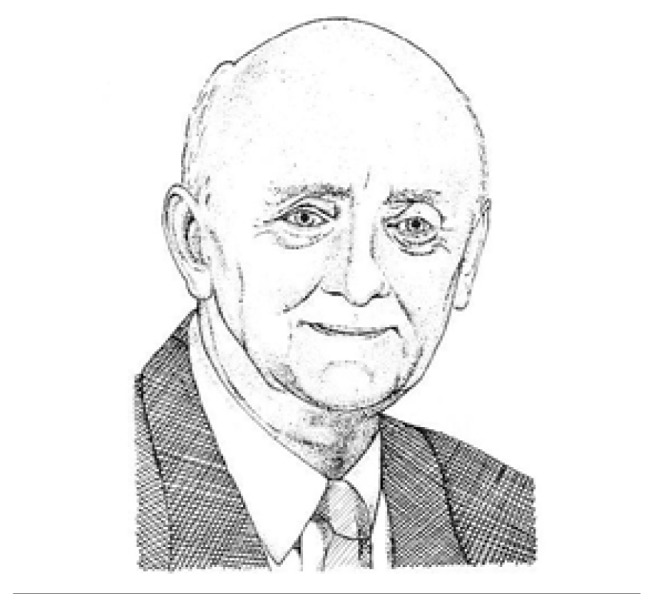
Loran Archer received his initial training as a vocational rehabilitation counselor. Prior to coming to NIAAA, he served in various positions in California’s alcohol programs, including director of California’s alcoholism prevention and treatment programs. Mr. Archer worked at NIAAA from 1977 up to his retirement in 1993.

***Archer****: *For one thing, the Institute has been very fortunate in terms of outside support. Several secretaries of the Department of Health, Education, and Welfare (now called the Department of Health and Human Services) were very interested in alcoholism. Most of them were not in positions to help the Institute much fiscally, but they were able to provide political support. The same applies to the surgeon generals. Working with some of them probably was one of the most fortunate experiences I have had, particularly with Dr. Everett Koop, who was very innovative, aggressive, and willing to commit his time and effort to the alcohol field.

Another very important factor for the Institute’s success is that NIAAA always has been willing to keep a very broad view of all the aspects of alcohol problems instead of focusing on any single biological or social model of alcoholism. The Institute has supported research in areas from molecular biology to macroeconomics. This versatility has allowed, and hopefully will continue to allow, the Institute to provide good research information on which public policy can then be based. This has been true for public policy issues related to biological or medical aspects of alcoholism, such as questions on liver transplants; for issues of health care utilization; and for issues of health insurance. NIAAA was at the forefront of the initial effort to provide health insurance coverage for alcoholism. And if and when a national health insurance system ever is established, NIAAA hopefully will have the research information to assist in devising a public policy that includes adequate alcoholism prevention and treatment.

***AH&RW:*** Lately, much of the Institute’s focus appears to be on the molecular biology aspects of alcoholism.

***Archer:*** I don’t think molecular biology is necessarily a focus of NIAAA. Molecular biology is exciting in every biomedical area, whether it is cancer, arthritis, cystic fibrosis, or alcoholism. Consequently, molecular biology gets a lot of press coverage. But when you look at what the Institute actually is doing, there are many other projects going on that will continue to be important. People do not get as excited about some of the other aspects of alcoholism, but then, molecular biology too may not be as exciting in a few years.

At the moment, we are going through a period where knowledge about genes involved in alcoholism and other diseases is developing very rapidly. The danger is that we get so preoccupied with the genetics that we assume that genes are the total cause of alcoholism. We have to remember that genes are only one aspect of alcoholism and that the environment also plays an important role. I think the Institute very successfully has kept a balanced view and has emphasized that although there may be aspects of alcoholism that are genetically determined, the environment also is a major contributor.

***AH&RW:*** In your opinion, how have the developments and scientific breakthroughs in the alcohol field affected the individual alcoholic?

***Archer:*** The alcohol field, like many others, changes only slowly. There is no dramatic jump, no fast change in paradigms. But when you look back 10 or 20 years, you notice that there are little changes. It is much like what is going on with smoking. People watching movies from the 1930’s are struck by what seems to be very unusual behavior, namely many people smoking. We notice it because there has been a major change in behavior. The same is happening with alcohol. People who have been involved with Alcoholics Anonymous (AA) and other organizations for a long time say that in the 1930’s, they felt a public pressure to drink. Today public attitudes toward drinking have changed, and abstinence is an acceptable behavior. That has been one of the most important changes that I have seen happen, and the Institute has been at the forefront of this development.

After its creation, the Institute had the main responsibility for establishing the infrastructure for treatment and prevention programs at the State and local levels. After that was done very successfully, the responsibility for these programs was transferred to local communities and the States. NIAAA also took the lead in establishing programs with business, such as employee assistance programs, which identify people with alcohol problems early, and in getting alcoholism treatment included as a part of general health insurance. These developments have made a major difference for the individual alcoholic.

***AH&RW:*** What do you consider the most important objectives for NIAAA for the next 25 years?

***Archer:*** I think that we still have major needs concerning adequate prevention and identification of alcohol problems. We still have large numbers of individuals who have alcohol-related problems, and we need to do a better job in preventing these problems. The effectiveness of treatment will continue to improve as we learn more and develop innovative approaches, but I think the greatest need for improvement will be in the area of prevention. We need better information about who is most highly at risk of becoming alcoholic. Right now we are using very broad measures of high risk. For example, we have known for a long time that the highest risk group is family members of alcoholics, but that is such a rough measure; many children of alcoholics never become alcoholic themselves. So far we do not know how to identify individuals at risk more accurately. But we hope to develop some criteria out of the major ongoing studies, such as the Cooperative Agreement on Genetics of Alcoholism (COGA) or the large-scale genetic studies.^4^

John R. DeLuca, now deceased, was director of NIAAA from 1979 until 1981. When once asked for his reflections on NIAAA’s activities during its early years, DeLuca replied,

There have been a number of specific advances since 1971, but perhaps the most significant achievement is the greater maturity that now guides this public health effort. This is apparent in the greater depth and breadth of understanding of those within the field, and in a more sophisticated understanding of alcoholism by the public. This maturity has brought us a deepened appreciation of the complexity of the disease, and has enabled us to discard many myths that once affected our responses to it. The veil of shame that in the past surrounded alcoholism is being lifted, allowing us to see it as a treatable disease and not as a failure of character.

(Deluca, J.R. Editor’s Forum. *Alcohol Health & Research World* 12(4):268–277, 1988)

***AH&RW:*** What did your work at NIAAA mean to you personally, especially since you were with the Institute for so many years?

***Archer:*** For me, the alcohol field was exciting and a lot of fun to work in. I have had the opportunity to work with some very interesting people, from Dr. Hollister in the California Department of Public Health to all the different NIAAA directors. Each one of them was a different personality and enjoyable to work with.

The work also gave me a lot of personal satisfaction. I saw treatment improve and become more available. I saw a change in society, which I thought the Institute contributed to, toward making recovery more acceptable. I saw some positive aspects in my own personal knowledge of people who have recovered from alcoholism. And now that I have retired, I have the time and the freedom to work in an emeritus type of situation on some questions that I have always had. It was rewarding being in the administration, but it did not give me the opportunity to really delve into some of the research questions. I hope that some of the things my colleagues and I are looking at now can have some impact and usefulness in the public policy arena. We still need to know more about many areas that will affect public policy, such as the economic aspects of alcoholism, questions about raising taxes [on alcohol], and questions on high-risk individuals.

## William E. Mayer, M.D

### Director, 1982–1983

***AH&RW:*** Dr. Mayer, can you tell me a little bit about how you got started in the alcohol field?

***Mayer:*** I was trained in psychiatry, finishing my senior residency at the Langley Porter Neuro-Psychiatric Institute in San Francisco in early 1950. At that time, psychiatry was still not widely used by the general public, and people were pretty uneasy about it. Those of us who wanted to start a private practice had to rely entirely on referrals from senior faculty members of medical institutions. I had a good relationship with several senior faculty members, and they started sending me patients.

In my practice, I discovered a couple of things right away. One was that many, if not most, of my patients had serious alcohol problems. And these were people who were working, who were making a living, who were socially acceptable, and so on. The second observation was that the patients did not seem to improve with the psychoanalytically based psychotherapy that I was doing. It didn’t dawn on me then that what we had been taught until then—that alcoholism was a character and behavior disorder, that it represented deficient moral strength, or that it was a manifestation of an underlying psychiatric problem—perhaps was not correct.

That was the beginning of my involvement with the alcohol field. In the mid-1960’s, I worked for the California community mental health system, which in those days was very progressive, and I developed a community mental health program in Eureka, CA. Among our patients were many alcoholics, whom we treated not as if they were neurotic or had a character disorder but as if they had a chronic physical disease. We discovered that if we were genuinely nonjudgmental, the alcoholics developed a trustful relationship with us, kept coming back, and had longer and longer periods of abstinence. Through those experiences, I became utterly convinced, which I am to this day, that once you have developed the disease alcoholism, you can never, ever, under any circumstances, consume any kind of alcohol without serious risk of harm. We taught that to the patients, and we were extremely successful with the program.

Subsequently I became first the chief deputy and then the director of the California Department of Mental Hygiene and finally the overall health commissioner in California. In the latter position, I saw a wide variety of approaches to the treatment of alcoholics in the community, most of which were not very effective. I increasingly became convinced that the only demonstrably successful long-term management and the only way to maintain a normal life for most alcoholics was to be very active in AA, and I continue to believe that today.

Later I spent 3 years in Germany running an alcoholism treatment facility located at an army medical center. There we treated a vast array of people with alcohol problems, including people in extremely responsible positions, even an officer in charge of a nuclear artillery unit. I also instructed every new commanding officer who arrived in Europe about alcoholism and how to deal with it successfully. Our program was enormously successful and had tremendous support from the military high command. That was an absolutely superb experience, probably the most rewarding professional experience of my life.

After I returned to this country, President Reagan asked me to become administrator of ADAMHA. The position was somewhat tricky, because the three Institutes (NIAAA, NIMH, and the National Institute on Drug Abuse [NIDA]) were not really very closely associated with one another, intellectually or philosophically. But I did have a wonderful year-and-a-half at ADAMHA.

***AH&RW:*** What did you consider as NIAAA’s main mission during your tenure as the Institute’s director?

***Mayer:*** One thing I always have felt strongly about is that alcoholism is a serious organic disease, which is obvious, considering the liver or brain damage involved. I was convinced that being an alcoholic was purely accidental and that personality, character, intelligence, or culture determined the development of alcoholism no more than it did the development of disorders such as diabetes or chronic heart disease. I also felt that the field of alcoholism needed legitimacy among the rest of the medical profession. As long as it didn’t have legitimacy—and it still doesn’t in many areas—it was very difficult for alcoholic patients to get the treatment and long-term care they needed. Consequently, I tried very hard to convince people in government, in the bureaucracy, in the medical profession, and among the patients themselves that alcoholism was a legitimate disease. I told them that alcoholism was treatable, although as far as we knew not curable (but neither are diabetes or hypertension), and that we could manage it like other chronic diseases. That was my main mission as NIAAA director and as ADAMHA administrator.

**Figure:**
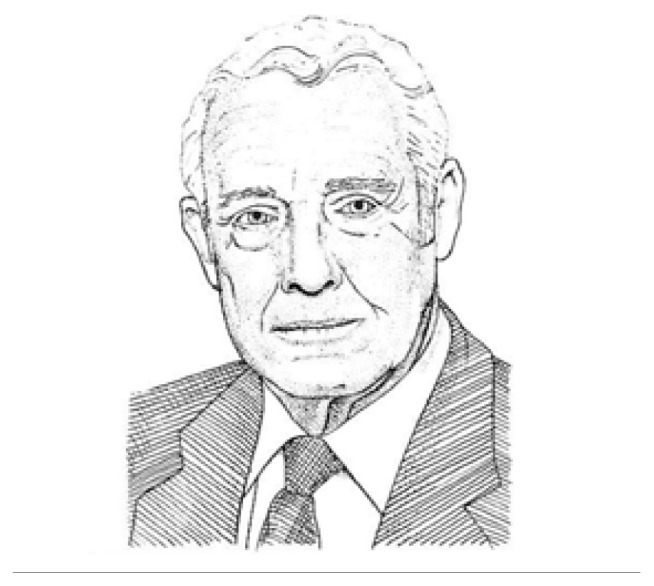
Before being appointed administrator of ADAMHA in 1981, William E. Mayer, M.D., held several high-level health care posts in California. Concurrent with being ADAMHA administrator, Dr. Mayer served as director of NIAAA. He subsequently was appointed Assistant U.S. Secretary of Defense for Health Affairs and later served as director of the Department of Mental Health in California before retiring in 1992.

***AH&RW:*** Where do you think NIAAA had the strongest impact in the alcohol field—in research or in public policy?

***Mayer:*** I think it was probably in research, but I hasten to add that its support of treatment programs was an absolutely crucial achievement. Lurking in the background of all discussions about alcoholism was the belief by many people in the power structures that if you were an alcoholic, you were a bum and really didn’t deserve good treatment. Alcoholics existed and something had to be done about them, but nobody really wanted to be bothered. Breaking down those old stereotypical and prejudicial beliefs, especially among influential members of Congress and the Administration, was terribly important, and I think much of what NIAAA has done has helped along those lines. These efforts have to continue because I think that alcoholism and its treatment are still not established parts of medicine.

But I also consider the research very important and successful, especially with respect to genetics, to the familial incidence of alcoholism. For example, researchers at the University of California, San Diego, turned up some fascinating linkages between alcoholic fathers and sons who had alcohol problems. Unfortunately, a lot of the genetic and familial research is considered by some of the more purist researchers as a kind of “soft” social science research. But I think there has to be an element of that in alcohol research, because alcoholism does partly manifest itself in behavioral and social terms, and some of the research in that area has been extremely important. In addition, the research conducted on alcohol metabolism and on FAS was fabulous.

Finally, an achievement that I take a great deal of pride in was in addressing the problem of alcohol abuse and the rapidly rising rate of deaths from alcohol-related automobile crashes among adolescents. NIAAA and the NCA started a program throughout the United States in which high schools that had programs designed by and for their students to diminish alcohol abuse competed against each other. The best program in each State was selected, and the 50 winning teams convened for a conference in Washington, DC. I think this program was a marvelous example of what Government can and should do, of how Government can start focusing public attention in a constructive way on a growing problem. And the problem has steadily diminished since that time: Fewer adolescents are being killed in alcohol-related accidents, especially on graduation night.

***AH&RW:*** What do you consider the most pressing problems for the next decades in the alcohol field?

***Mayer:*** Early identification. The susceptibility to alcoholism probably can be identified much earlier than we can do at this time, in adolescents perhaps even before they drink any alcohol. There must be biological markers by which it will be possible to predict heightened susceptibility to alcoholism. And to the people at risk, we have got to say, “Look, it is not your fault; you are not necessarily destined to become alcoholic, but you are 10 times more likely than your friend. Here is what you need to be aware of.” We have got to develop a special training and education program for these people; they should be the principal recipients of our best prevention efforts.

I also think NIAAA needs to be strongly and publicly involved in the study and prevention of the consequences of alcohol abuse—not alcoholism, but alcohol abuse. Some really serious problems exist among people who abuse alcohol. An incredible percentage of people who commit crimes do so while drinking, and a huge amount of family violence and street violence is connected with alcohol. This association of alcohol with violence, family disruption, crime in general, accidents, general health problems, and loss of productivity must be addressed very aggressively.

When looking at these problems, however, there is a danger of getting alcohol abuse mixed up with addictions to other drugs. Of course some people will abuse any drug they can get their hands on. But I believe that there is a world of difference between alcoholism as a disease and the use and abuse of and dependency on most other drugs. Therefore, I do not like grouping alcohol and other drugs together, and I do not think that measures designed to help manage the chronic disease of alcoholism necessarily can be applied successfully in the management of addiction to other drugs. A high percentage of addicts and drug abusers have profound characterological problems, whereas alcoholics as a group include many people who have demonstrated successful life patterns. I think in most cases, alcoholics and drug abusers are different populations and should be addressed separately.

***AH&RW:*** What specific characteristics have allowed NIAAA to be so successful in advancing the alcohol field?

***Mayer:*** What is unique about NIAAA is that the Institute has managed, albeit with varying success from time to time, to continue an advocacy and public activism, with respect to educating the public about alcohol, that has remained nonmoralistic, and that, I think, has been extremely healthy. I also think that the Institute has been very good about publicizing research results. This information effort needs to continue, because alcoholism still is seen as a behavioral problem by a small but significant portion of the public.

***AH&RW:*** Dr. Mayer, how would you summarize your tenure at NIAAA?

***Mayer:*** It was an enormously rewarding professional experience that contributed greatly to my understanding of a huge population of patients who need help, who often respond to help, who can be managed like any chronically ill patients, and who deserve the very best medically and psychiatrically that we can give them.

## Robert G. Niven, M.D

### Director, 1983–1985

***AH&RW:*** Dr. Niven, can you describe briefly why you got involved in the alcohol field?

***Niven:*** What initially sensitized me to addiction problems was having a friend in high school whose parents both were alcoholics and another friend in medical school who developed and ultimately died of serious drug-dependency problems. Later, as a resident in internal medicine at the Mayo Clinic, I had to do a rotation in the psychiatry department. There I began to see that alcoholics and drug addicts could be treated successfully. During my rotation, the clinic’s alcoholism program held a reunion of its “graduates.” I was very impressed with, and intrigued by, how well these people were doing in their lives and how pleasant, happy, and responsible they were. They were very different from the stereotypical view of alcoholics. I later changed my specialization from internal medicine to psychiatry. I often worked in chemical dependency treatment units and found the patients a very interesting group to work with. Subsequently, chemical dependency became my subspecialty for my entire psychiatric career.

***AH&RW:*** Do you think that as a clinician, you had a different perspective on NIAAA and your position than directors who came from a more research-oriented background?

***Niven:*** Perhaps I did have a slightly different perspective than somebody with a pure research background. But I strongly believe that ultimately, research will provide the answers to alcohol problems. Being a good clinician involves utilizing the best scientific data available and pertinent to the clinical issue at hand. It also involves applying the knowledge base both to a given patient’s situation and to issues beyond the individual patient, for example, in the prevention field. Consequently, I believe in NIAAA’s research mission and consider it absolutely critical.

When I became NIAAA director, the Institute was still undergoing its transition from a prevention and treatment focus to a research focus. One of the things I could accomplish during my tenure was to strengthen support for NIAAA’s research mission in Congress and the Administration. At that time, nonresearch groups in the alcohol field, such as the lay public and some prevention and treatment organizations, had mixed feelings about whether NIAAA should be involved in research. They saw funding for research gradually increase, whereas funding for treatment services declined. That caused some animosity and skepticism toward NIAAA in Congress and among the non-research constituency. However, we were able to overcome these negative perceptions, to strengthen our research activities, to increase our budget, and to attract more top-quality researchers to the intramural and extramural programs. The involvement of these researchers and of other nonresearch organizations helped the Institute to grow at a time when skepticism still persisted regarding whether alcoholics even were worthy of treatment and research.

***AH&RW:*** What do you consider were NIAAA’s primary achievements during the past 25 years?

***Niven:*** I think that the Institute’s primary achievement was—through the research, treatment, prevention, and epidemiologic components of its activities—to build a scientific knowledge base of information about alcoholism. NIAAA also helped establish the concept among a significant portion of the public that alcoholism is a major health problem, even a public health problem. The Institute demonstrated that many alcoholics were treatable and that some of the alcohol-related morbidity and mortality, as well as some of the horrendous economic costs that are consequences of alcohol and other drug abuse, could be prevented.

These changes may not have been very glamorous, but they were critical. In my just over 20 years in the field, I have seen enormous developments in the acceptance—by the public, the Government, and Government agencies—of alcohol-related problems as a group of treatable and preventable problems. Society still is not where it should be with respect to the overall perception of alcoholism; there still is much work to do. In fact, antialcoholism and antidrug abuse treatment forces seem to be gaining ground lately. Nonetheless, I think we have made much progress. But although NIAAA was a major player in bringing about these changes, it also is important to recognize that it was not the only player; other groups and organizations also contributed significantly to these efforts. The continued collaboration among such groups is essential if we are to continue making maximal progress.

***AH&RW:*** As a clinician, do you think that all the recent scientific advances already have affected the treatment of the individual patient?

***Niven:*** I think that although alcohol research is no longer in its infancy, it still is in its childhood. Some neurobiological or genetic studies still are in the early stages of development and have not advanced sufficiently to be applicable to the general treatment of alcoholics. However, within the next decade, researchers and clinicians should be in a position to advance significantly the treatment applicability of these technologies for a much broader patient population.

***AH&RW:*** What do you consider the most pressing alcohol-related needs that the alcohol field will face in the future?

**Figure:**
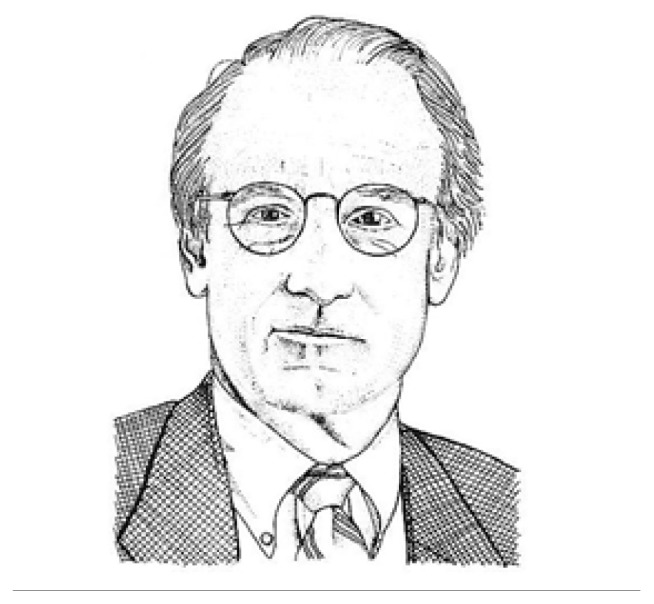
Before being appointed director of NIAAA, Robert G. Niven, M.D., served as director of the Mayo Medical School Alcohol and Drug Abuse Teaching Program and of the Mayo Clinic Adolescent Drug Abuse Service. Dr. Niven currently is the medical director of the Chemical Dependence Program in the Department of Psychiatry at Harper Hospital in Detroit, Michigan. In addition, he is an associate clinical professor at the Wayne State University School of Medicine in Detroit.

***Niven:*** First, I think that we need to develop better pharmacological agents to minimize alcohol-induced pathology. By minimizing or reversing the physiological impact of alcohol and other drug overdoses and intoxication, we can significantly decrease alcohol-related morbidity and mortality.

Second, we need to identify the genes responsible for or involved in developing tolerance and in developing the disease of alcoholism. We also need to identify the genes responsible for making particular subgroups of alcoholics more or less susceptible to certain complications of alcoholism, such as Wernicke-Korsakoff’s syndrome, liver disease, or pancreatitis. Progress in this area would enable us to identify people at risk for these disorders; could open the doorway for more specific interventions, including pharmacological measures; and would ultimately allow us to make another giant stride in reducing alcohol-related morbidity and mortality.

Third, we still have a long way to go in terms of preventing alcohol-related problems, although we certainly have made advances in that area. For example, it was very encouraging to see that alcohol consumption declined for several consecutive years and that we were making some real progress in diminishing alcohol-related automobile crashes. Unfortunately these developments seem to have leveled off recently.

Another prevention area that needs much more attention is the impact of parental alcoholism and other forms of drug dependence on the behavioral problems of children, in addition to the clinical effects of FAS and the increased risk for alcoholism that children of alcoholics have. In my clinical practice, I constantly see how alcohol abuse contributes to both domestic violence and the abuse and neglect of children. These problems still are seriously underestimated, underdiagnosed, and undertreated even when they are recognized. We need more education about the consequences of parental alcohol abuse—for example, in the school systems; among pediatricians; or in agencies focusing on children, such as child protective services.

In summary, I think we need to increase the research on more effective prevention strategies. I am convinced that alcohol abuse still is a major underdiagnosed and undertreated health problem and a major contributor to health care costs. But I firmly believe that many current deficiencies can be changed for the better.

Another issue that intrigues me as a clinician is the individual variability that exists in some alcohol-induced pathology. I have seen people who drink 1 pint of alcoholic beverages every day for 5 years develop liver cirrhosis, whereas other people drink more than that for 20 years and still have perfectly normal liver functions. There must be an explanation, likely genetic, for these individual differences, and we need more research to determine what it is. Such research also should include the analysis of the chemicals in alcoholic beverages other than alcohol itself.

***AH&RW:*** Ten years ago, you said that you did not think that society could eradicate alcoholism in your lifetime as it has other diseases. Has the scientific progress since then made you change that opinion?

***Niven:*** I still do not believe that substance abuse, particularly alcohol abuse, will ever be eradicated completely. After all, alcohol has been around since antiquity and is produced so readily from a variety of sources. But I do think that we can significantly reduce alcohol-related pathology and alcoholism’s economic burden on society. It will be a slow process, because there are powerful worldwide economic interests involved in producing and selling alcoholic beverages. But if we could reduce the alcohol problem to the level of, for example, the heroin problem, I think most people in the field would be elated. As a clinician involved in chemical-dependency treatment, I certainly am not trying to denigrate the significance of the heroin problem in our society, but it is just many, many times less severe than the alcohol problem.

***AH&RW:*** What kind of an impact did your tenure at NIAAA have for you on a more personal level?

***Niven:*** As with any other job, there were both good and bad experiences. It was a fascinating, albeit at times an incredibly frustrating, job. It educated me about the political processes connected with research. Before I became NIAAA director, I never really fully appreciated the significance and the broad public health impact that an agency such as NIAAA could have. For example, after I had been at the Institute for only 1 month, the health care funding concept of diagnosis-related groups (DRG)^5^ was implemented. As a clinician, I realized that the DRG limits for alcoholism treatment did not reflect the reality of treatment services and that the data on which the limits were based were seriously flawed. I think it would have devastated the treatment field if we had not been successful in challenging the DRG plans. Therefore, I felt very gratified personally when we were able to stop these plans (while we developed better data) in an enormous team effort of NIAAA staff, of ADAMHA staff under ADAMHA administrator Robert Trachtenberg, of people at NIDA and NIMH, and with strong support from the treatment community and from Betty Ford.^6^

Another very personal advantage was that the position allowed me to meet many great people—researchers; the staff at NIAAA; administrators, such as Robert Trachtenberg; heads of other Institutes, such as William Pollin at NIDA and Herbert Pardes at NIMH; or people with NCA, the Children of Alcoholics Foundation, and other organizations. These personal contacts were very rewarding, and I value them to this day.

I also personally felt very pleased that I was able, with a few other psychiatrists, to facilitate an increased awareness of alcohol problems and the need for action to deal with them in organized psychiatry.

## Enoch Gordis, M.D

### Director, 1986–Present

***AH&RW:*** Dr. Gordis, how did you first become involved in the alcohol field?

***Gordis****: *It is amazing how fate propels you into things that you never anticipated, how you really can’t plan your future. Starting in 1961 I had been working in Dr. Vincent Dole’s laboratory at the Rockefeller University in New York City. During the 1950’s it was a leading laboratory in the study of fat metabolism, and I started to work in that area. Around that time, however, Dr. Dole was invited to replace the chairman of a meeting on New York City’s drug problems. At this city-wide leadership conference, Dr. Dole discovered that every participant had opinions on the topic, but nobody had any data. To him, that was not the way to deal with such a public policy issue. Consequently, he sought and got permission from the president of Rockefeller University to study heroin addicts at the university’s hospital, a project that eventually led to the development of methadone maintenance treatment for these patients.

The intellectual focus in Dr. Dole’s laboratory gradually shifted from fat metabolism to addiction, which was a very different kind of intellectual challenge. Consequently, when I completed a project in fat metabolism in the mid-1960’s and was looking for a new avenue to explore, I began to consider the alcohol issue. Dr. Marie Nyswander, a colleague in the laboratory, introduced me to a close friend of hers, Dr. Ruth Fox, who was a physician in New York City and one of the pioneers for the inclusion of alcoholism treatment into medical practice. Dr. Fox had introduced the alcohol-sensitizing medication Antabuse^®^ from Denmark and prescribed it to many clients in her practice. Thus, while I was still at Rockefeller University studying animal models and alcohol withdrawal, I also began to work with Dr. Fox in her practice to learn her approach to treatment.

That was the “accident” by which I entered the alcohol field. As a youngster, I hardly would have predicted that ultimately as an adult, I would study problems related to alcoholism, but that is the kind of surprise that life sometimes has in store.

In 1971 the opportunity arose to establish an alcoholism program at the city hospital in Elmhurst, NY. The funds had been provided to the hospital’s psychiatry department by the State, but because at that time there was still a strong disdain for alcoholics, nobody wanted to start the program. I think some of the members of the department at the time felt that alcoholics and addicts ought to be put on some island and left there; a view, by the way, that unfortunately might find some sympathy in this country even today. So I established and administered the program in Elmhurst, where we treated some 15,000 patients during the next 14 years.

***AH&RW:*** What, in your opinion, were the primary achievements of NIAAA during the past 25 years?

***Gordis****: *One could list numerous important scientific insights concerning treatment, genetics, the development of animal models, or the importance of neuroscience. But I think what counts most is that NIAAA showed that research can make an important contribution to solving the alcohol problem, that in fact it is an indispensable element to solving the alcohol problem. Alcoholism treatment can be approached with the same scientific rationale and style as other areas of medicine. It has stopped being a vaguely formulated problem and instead gradually has been brought into the mainstream of medical science. The individual findings are important, but the general recognition that alcoholism can be studied with the most contemporary tools of science is the contribution that stands out.

**Figure:**
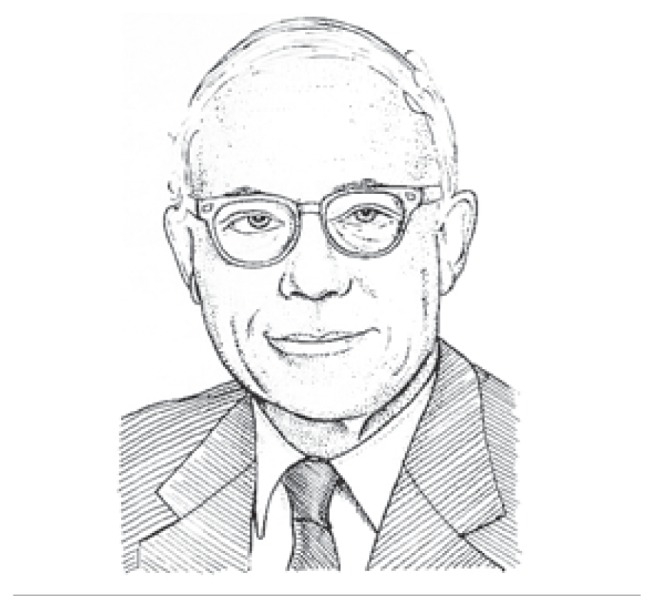
Enoch Gordis, M.D., originally was trained in internal medicine. He became involved in alcohol research during a 10-year stay at New York City’s Rockefeller University. Dr. Gordis subsequently founded the alcoholism treatment program at the City Hospital Center in Elmhurst, New York, and also was professor of clinical medicine at Mount Sinai School of Medicine in New York City. Dr. Gordis was appointed director of NIAAA in 1986.

***AH&RW:*** Do you think that the academic achievements in the alcohol field have translated into improved care for the individual patient or a changed perception of alcoholism by the public?

***Gordis:*** Scientific progress already has affected the patients and the public in some ways, and I think that it will continue to do so at a very accelerated pace during the next 10 or 15 years. Obviously, defining FAS has had a major public health impact. And the knowledge that a genetic component contributes to the vulnerability to alcoholism has been instrumental in forming the public’s perception of alcoholism as a disease. Also, in the last few years, the treatment community has begun to recognize the importance of measuring treatment outcome and conducting proper clinical trials using blinding, randomization, placebos, and so on. We are entering an era in which the treatment community will be affected heavily by the results of research, probably in the next 5 or 10 years. All these things are happening and are supported strongly by NIAAA.

***AH&RW:*** One of your interests is the interaction between science and public policy. Do you think that the recognition for the alcohol field in the public policy arena has improved?

***Gordis****: *The interaction between science and public policy has brought mixed results over all. For example, during the recent discussions of health care reform, the importance and value of alcoholism treatment clearly were recognized. The details are not as important at this point as the fact that alcoholism treatment would have been included in a reform package, whatever its form. In fact, I believe that in some places, it already is being considered a part of the mainstream reimbursement system without special caps or limitations. These are very positive developments.

There also is a greater respect now in Congress for the problems of alcohol abuse. Legislation has been passed that effectively demonstrates that change—such as the 21-year minimum age for alcohol use, with its impact on drinking and driving. The rights of alcoholics also were incorporated in legislation during the last 20 years.

I believe that alcohol research served as the foundation for these changes. The ongoing research activities saved us from attacks on the concept of alcoholism as a disease and helped shatter the idea of alcoholism as “willful misconduct.” This probably was brought about not by any single conclusion but by the general feeling that the disease alcoholism is a puzzle and that science is the way to solve it.

But there have been some setbacks as well. The alcohol field still does not command the same respect as other areas of medicine. Some people once again consider addiction just an excuse for misbehavior, not a cause. And I think that part of the discussion about alcoholism in relation to disability also has been retrogressive. Thus, the developments have been mixed, but overall they represent progress.

***AH&RW:*** What do you consider the big challenges for NIAAA in the next 25 years?

***Gordis:*** I believe that the direction that alcohol research is taking is excellent on all fronts. However, we still are a small field, considering the number of investigators involved, and funding is not readily available. Obviously it is preferable if an institute or a scientific group is large and diverse, because every field needs new ideas. However, new ideas often are proposed by young investigators who may not get a chance to pursue them. That is one of the challenges that we face. But I think that as alcohol research continues to evolve and mature, it will produce both interesting answers and new questions that can attract new people.

Our major policy problem is that we still have no significant lay constituency for alcoholism. Other medical disciplines have organizations, such as the American Cancer Society, that are initiated or managed either by the patients or their family members and that mount campaigns for funding to support research. That is not yet happening in the alcohol field, although some efforts are being made in that direction. The lack of a lay constituency is perhaps our primary obstacle as far as the public perception of alcoholism is concerned.

Another challenge for the future is to erase the old public misperception that although research may be very nice, we basically already know how to treat alcoholism effectively—all we have to do is get patients to attend AA meetings or therapy. In reality, however, it is quite the opposite. Alcoholism treatment only has limited effectiveness; many patients either do not receive treatment, do not like the treatment they receive, or relapse eventually. Although current treatment has a positive cost-benefit ratio, we still have so much more to learn.

***AH&RW:*** What makes alcohol research such an exciting field, and in which area does your main interest lie?

***Gordis****: *My relationship with the different areas of alcohol research is like a parent’s relationship with his children—they all are different but he loves them equally. Because of my training in internal medicine and my laboratory science background, I obviously have a persistent interest in that side of our work. But I believe that top people in any discipline have something to teach us, and it is a mistake to cling tightly to one’s own area of expertise and decide that that is the only way for the field to go. Therefore, areas such as the psychosocial sciences also are very important for many reasons. Besides, it is very hard to predict at any given moment whence the next major breakthrough will come. Thus, whether it is molecular biology and genetics, the economics of alcohol consumption, or anything in between (such as treatment, prevention, or toxicology), I consider all these areas important.

Why is alcohol research so attractive? Alcoholism affects so many facets of society that one can make a contribution to the field no matter what one’s background is. Whether researchers study treatment and prevention or analyze the molecular biology of some nerve cell receptor, they all are doing something that is vital to the field. There are not many fields in which that is true, and I think that is part of the appeal and attraction of alcohol research for many people. What also makes the field, and my position as Institute director, very enjoyable is that the challenges and questions in our science are extraordinarily interesting and solving them will benefit the public immensely. Therefore, one feels that what one is doing is intrinsically worthwhile.

Another challenging aspect of alcohol research, which also is true in many other areas of medicine, is that its fundamental question is still not answered. That question is, “What is the relationship between this substance and this individual person that enables the substance to dominate the person’s life to the exclusion of everything else?” That is what we do not understand and what we are trying to learn. Once we understand this relationship, there is hope that we can disrupt it.

## Footnotes

1 Senator Harold Hughes was chairman of the Subcommittee on Alcoholism and Drug Abuse in 1969/1970, which drafted Public Law 91–616. This law, among other things, established NIAAA. The Senate approved the bill on August 10, 1970, and the House of Representatives followed on December 18, 1970. President Richard Nixon signed the bill into law on December 31, 1970.

2 Marty Mann was the first female member of Alcoholics Anonymous. She also founded the National Committee for Education on Alcoholism, which later became the National Council on Alcoholism.

3 ADAMHA was the umbrella organization for NIAAA and two other Institutes of the National Institutes of Health from 1973 to 1993.

4 COGA is a multidisciplinary study involving seven research institutions throughout the country that is attempting to elucidate genetic components of the vulnerability to alcoholism. Other genetic studies funded by NIAAA focus on brain functions and alcohol metabolism both in humans and in animal models.

5 In the health care compensation model of diagnosis-related groups, hospitals were paid a fixed amount for the treatment of various categories of diseases, based on the calculated length of stay of patients with these diseases. The goal was to reduce above-average hospital stays and thus control health care expenditures.

6 The wife of former U.S. President Gerald Ford.

